# Balancing Dietary Vitamin D_3_ and Phosphorus Improved Growth, Mineralization, and Fecal Characteristics of Atlantic Salmon (*Salmo salar*) Post-Smolts in Land-Based Aquaculture

**DOI:** 10.1155/anu/4044914

**Published:** 2025-11-17

**Authors:** Vegard Øvstetun Flo, Jon Øvrum Hansen, Christopher Hawes, Tomé Silva, Ashleigh Currie, Jannicke Vigen, Odd-Ivar Lekang

**Affiliations:** ^1^Cargill Aqua Nutrition, Cargill, Bergen, Norway; ^2^Department of Mechanical Engineering and Technology Management, Norwegian University of Life Sciences, Ås, Norway

**Keywords:** Atlantic salmon, cholecalciferol, mineral, NQC, phosphorus

## Abstract

Metabolic responses to different dietary levels of vitamin D_3_ and available phosphorus (avP) was investigated in cultured Atlantic salmon (*Salmo salar*). The study was carried out in triplicate with a 2-level full factorial design with a center point and three additional points within the design space. Over a 17- week period at the initial weight of 453 ± 9 g, salmon reared indoor on land, were fed diets containing different levels of vitamin D_3_ and avP. In summary,growth performance, measured as specific growth rate (SGR), improved when either vitamin D_3_ or avP was increased independently, but declined when both were elevated excessively. Bone mineralization was maintained at intermediate vitamin D_3_ inclusion levels, though high vitamin D_3_ reduced bone ash. A diet containing 0.63 mg/kg vitamin D_3_ and 0.70% avP supported growth and mineral retention. A significant reduction in fecal soluble phosphorus when vitamin D_3_ was increased in the diet, suggests that management of vitamin D_3_ can contribute to improved waste control and reduced environmental load for phosphorus. Finally, the study highlighted that today's commercial dietary inclusion of vitamin D_3_ can result in lower vitamin D_3_ accumulation in muscle of land-based farmed Atlantic salmon compared to wild Atlantic salmon.

**Summary**

• Increased dietary levels of vitamin D_3_ from 0.2 to 1.12 mg/kg at available phosphorus (avP) levels of 0.70% increased specific growth rate (SGR) but reduced bone ash content in Atlantic salmon (*Salmo salar*).
• Fish reared in land-based aquaculture facilities without natural light exposure accumulated below average levels of vitamin D_3_ in muscle, when fed diets containing normal commercial concentrations of vitamin D_3_.
• Increasing dietary vitamin D_3_, can reduce the fecal waste of soluble phosphorus and Zn.

## 1. Introduction

The fat-soluble vitamin D and its various metabolites are crucial for maintaining the health and vitality of fish, and play a key role in regulating calcium (Ca) homeostasis [1]. The interactions between vitamin D, Ca, and P are essential for various biological functions in fish, including skeletal development and growth, where mineral homeostasis is regulated by the endocrine system through hormones, such as stanniocalcin [2], calcitonin [3, 4], prolactin [5, 6] and parathyroid hormone-related protein [7, 8]. These hormones influence the absorption of Ca and P from the intestine, their mobilization from bone, and their reabsorption or excretion via the kidneys, thereby maintaining systemic mineral balance.

Studies have confirmed a strong correlation between gastrointestinal disorders, vitamin D deficiencies, and fish bone diseases [9–11], emphasizing the critical link between vitamin D and bone-forming minerals like Ca, P, and magnesium (Mg) [12]. While some studies have shown species-dependent responses to vitamin D metabolites in mobilizing P for freshwater eel (*Anguilla Anguilla*) [13] and common carp (*Cyprinus carpio*) [14], other studies do not indicate any specific links between vitamin D concentrations in diet and utilization of P [15, 16]. Even though concentration of vitamin D in the diet seems to have little effect on vertebrate and whole body concentrations of P in fish, there are indications that Ca, P, and Mg concentrations in the skin and scale of rainbow trout (*Oncorhynchus mykiss*) increase with increasing vitamin D in the diet [17]. Vitamin D has also been shown to have positive effects on fish health parameters, such as promoting resistance to oxidative stress and inflammation in yellow catfish (*Pelteobagrus fulvidraco*) [18], modulating the innate immune system in seabream (*Sparus aurata*) [19], and improving the skin mucosal barrier against pathogens in zebrafish (*Danio rerio*) [20].

In aquaculture, fish primarily obtain vitamin D as vitamin D_3_ (cholecalciferol) from their diet [21], with fish oils being a potent and primary source of this essential nutrient, and therefore, used in diets for farmed Atlantic salmon (*Salmo salar*) [22, 23]. Dietary requirements of vitamin D and available P (avP) for salmonids ranges from 0.02–0.04 mg/kg of feed and 0.6%–1.0% of the diet, respectively [24]. In addition to the dietary source, rainbow trout and Mozambique tilapia (*Tilapia mossambicus*) have shown an ability to synthesize vitamin D_3_ in the skins from a photochemical precursor, 7-dehydrocholesterol (7-DHC) [25–27], suggesting an alternative route for vitamin D synthesis. However, vitamin D_3_ synthesis under natural light conditions in fish remains uncertain [1].

Interestingly, vitamin D_3_ concentrations in the filet of farmed Atlantic salmon may suggest a link to light exposure. More specifically, farmed Atlantic salmon generally contain lower levels of vitamin D_3_ in the filet compared to wild salmon, with averages ranging from 2.9 ± 0.7 to 9.5 ± 0.7 µg/100 g [28]. This variability is relevant for the industry, as vitamin D_3_ is a key nutritional selling point for human health [29], and deficiency may lead to muscle tetany and “droopy tail” in fish [11, 30]. While the dietary requirement for vitamin D_3_ is relatively low, the European Union permits inclusion levels up to 1.5 mg/kg in aquafeeds [23], providing a wide regulatory margin for nutritional optimization.

The significant technological development in the Norwegian farming industry has enabled salmon to be reared to harvest size in indoor recirculating aquaculture systems (RAS) and hybrid flow-through systems. These systems often lack natural light, and it has been suggested that fish grown indoors may accumulate even lower levels of vitamin D_3_ compared to those reared in open pens [31]. This raises the need to explore how dietary vitamin D_3_ can be strategically adjusted within EU limits, to ensure adequate tissue deposition and support biological functions for salmonids reared in land-based farming systems. Previous studies have shown that increasing dietary vitamin D_3_ while reducing P can decrease fecal P emissions in rainbow trout [32]. This is of importance since RAS has shown to accumulate a range of minerals, including Ca and P [33, 34]. Therefore, further exploration of vitamin D's role in regulating mineral homeostasis may be important not only to support fish physiology, but also to inform treatment needs and potentially enhance water purification capacity in RAS [35]. As RAS technology continues to expand, partly due to its waste reduction capabilities and general alignment with stricter environmental regulations, nutritional strategies that reduce P discharge are increasingly important [36–42]. The interplay between dietary vitamin D_3_, mineral retention, and system-level accumulation suggests that vitamin D_3_ may serve not only as a nutritional regulator but also as a strategic lever for improving water treatment efficiency in RAS.

Vitamin D plays a multifactorial role, in Ca homeostasis, P metabolism, and fecal composition, with potential implications for both fish health and environmental impact. The present study was designed to test the hypothesis that dietary vitamin D_3_, within the European Union's maximum allowed inclusion of 1.5 mg/kg [23], in combination with varying P levels, can significantly influence growth performance, mineral retention, and fecal mineral excretion in Atlantic salmon reared without natural light. The objective was to determine optimal inclusion levels and ratios that promote growth and mineralization, while ensuring adequate vitamin D_3_ deposition in muscle tissue to meet nutritional expectations for human consumption.

## 2. Materials and Methods

### 2.1. Diet and Feeding

Seven diets were formulated based on a control diet containing commercially relevant P and vitamin D_3_ levels (MP-CD). Four diets were formulated with low–high P and vitamin D_3_ combinations. The three other diets were kept at commercial P levels (MP) while the total vitamin D_3_ level were set to 0.315 (MP-D03) and 0.827 mg/kg (MP-D08) and intermediate 0.637 mg/kg (MP-MD). To avoid vitamin D inclusion from fish oil and maintain better control, algae oil was used for securing satisfactory eicosapentaenoic acid (EPA) and docosahexaenoic acid (DHA) levels in the feed. Trial feeds contained yttrium oxide (0.2 g/kg diet) and were formulated using the Bestmix 5.0 software (Adifo Software, Olen, Belgium) with external oil mix calculations. To prepare experimental diets, feed ingredients were well grounded, mixed with water, and 2.5% of the rapeseed oil added during conditioning. The resulting blend were then processed using a twin-screw extruder (die diameter: 5.3mm; die length: 5.5 mm) and dried to target moisture content of approximately 6.5%. Algae oil with and the rest of the rapeseed oil was incorporated post-extrusion using a vacuum coater. The pellets were subsequently cooled, packed and stored at 5°C for further use.

All diets were produced at the Cargill Innovation Center (EWOS innovation, Dirdal, Norway) and formulated for 200 g Atlantic salmon. The formulation of the final 7 mm diets is presented in Table 1.

Feed was delivered continuously by an automatic belt feeder (Hølland Teknologi AS, Norway). The fish were fed to apparent satiation determined when excess feed was visually observed at the bottom of the tank. The excess feed, along with feces, were removed gently by a feed collector, designed based on the Guelph system principle [43]. Excess feed and feces were collected twice daily, in the morning at 07:30 and again at 14:30, and manually separated before the uneaten feed was weighed (wet and dry weight).

### 2.2. Proximate Analysis of Experimental Diets

Protein was analyzed by the Dumas principle using the Elementar Rapid Max N system (Elementar Analysesysteme GmbH, Langenselbold, Germany). Fat was analyzed by low-field nuclear magnetic resonance (LfNMR) scan using the NMR Analyzer Bruker minispec mq10 system (Bruker, Billerica, Ma, USA). Gross energy was analyzed by the Leco gross energy bomb calorimetry system (Leco, Geleen, The Netherlands). Moisture and ash were analyzed by the Leco TGA 701 analyzer (Leco, Geleen, The Netherlands). Vitamin D_3_ content was determined by high performance liquid chromatography in accordance with European standard (EN 12821:2009). Minerals were analyzed in accordance with DS/EN 13805:2014 and DS/EN ISO 11885 m:2009, for pressure digestion of foodstuffs intended for the determination of elements, with the inductive coupled plasma optical emission spectrometry technique (ICP-OES). For the measured proximate composition, see Table 2.

### 2.3. Fish Trial Conditions

The Atlantic salmon post smolt used in the study, originated from fertilized eggs supplied by Aquagen (Atlantic QTL-innOva SHIELD, Vestseøra, Norway) and were hatched and raised at Cargill innovation Center (EWOS innovation, Dirdal, Norway) prior to the experiment. The fish were pit-tagged (RFID), vaccinated (40–50 g) (Alpha Ject Micro 6, PharmaQ, Norway), and smoltified (at 70 g using a diet containing salt [EWOS ADAPT FLEX 40, Cargill, Florø, Norway]). With a mean initial weight of 325 ± 5 g, the fish were randomly distributed into 24 seawater (28 psu salinity) flow-through fiberglass tanks (1.5 m diameter and 0.94 m^3^ volume), aiming for 40 fish per tank at the start of trial diet feeding. After approximately 1 month of tank acclimation, the fish were 453 ± 9 g and were then fed the experimental diets over a 17-week period. The fish were housed under a 9 h light and 15 h darkness light regime with equal environmental parameters for all tanks. The experimental setup utilized 15 fluorescent lighting fixtures equipped with a 36 watt T8 tube. With a manual utilization factor of 0.8, average illuminance was estimated in accordance with the British zonal method to 96 Lx [44]. Water temperature averaged at 8°C (6–9°C) with an average oxygen saturation of 131 ± 10%sat (inlet water) and 90 ± 11%sat (outlet water) during the acclimation and the experimental feeding period.

### 2.4. Fish Growth and Performance

The 40 fish per tank were weighed and measured individually with PIT-tag identification on acclimation to the tanks (Day 0), at the start of trial diet feeding (Day 27), at intermediate weighing (Day 96), and after 119 Days of trial feeding (Day 146). The fish weight gain over the trial period were compared statistically between diets. Condition factor (K), specific growth rate (SGR), feed conversion rate (FCR), and protein efficiency ratio (PER) were determined as per the equations:

  $$\begin{array}{c} K=100\times \frac{\text{Weight},g}{{\left(\text{total length},\text{ cm}\right)}^{3}}, \end{array}$$

  $$\begin{array}{c} \text{SGR }\left(\%\text{/day}\right)=100\times \frac{\text{Ln }\left(\text{final biomass},g\right)-\text{ln }\left(\text{initial biomass},g\right)}{\text{time},\text{ days}}. \end{array}$$

The FCR was calculated as:

  $$\begin{array}{c} \text{FCR}=\frac{F}{w2-w1}, \end{array}$$

where w2 − w1 is the weight increment and F is the mass of the feed provided over a specific time period.

The PER was calculated as:

  $$\begin{array}{c} \text{PER}=\;\frac{w2,g-w1,g}{\text{Feed consumed},g\times \left(\frac{\text{protein in feed},\text{ \% }}{100}\right)}. \end{array}$$

### 2.5. Sampling and Analysis of Fish Tissue and Feces

All sampled fish in the study, were euthanized with a lethal overdose (over 250 mg/L) of tricaine mesylate (Pharmaq AS, Oslo, Norway). Standardized cutlet samples, known as Norwegian quality cuts (NQCs), were selected for tissue analysis due to their widespread use in commercial salmon production and the existence of an established vitamin D_3_ reference database maintained by the Norwegian institute of Marine Research [45]. The NQCs were cut with a vertical incision straight down from the rear part of the dorsal fin, and another vertical cut from the vent by the anal fin up towards the spine, in accordance with NS 9401 and as described by Johnsen et al. [46]. One fish from each of the 24 tanks was sampled at the beginning of the study (Day 27), and the NQCs were divided into two randomized pools (*n* = 12). Additionally, 24 fish were collected for whole body composition (Day 27), where one pool (*n* = 12) was analyzed while the remaining pool (*n* = 12) was stored frozen (−20°C). From the intermediate (Day 96) and final (Day 146) weighing, 10 fish from each tank were used for collection of NQCs, while 5 fish from each tank were collected for whole body analysis (Day 146). The NQCs from each fish were stored in individual plastic bags that were pooled together in a larger bag from each tank (*n* = 10) and frozen at −20°C before further processing. Storing NQCs in individual bags was done to avoid them from freezing together and potentially affect analysis. Average retention of selected minerals to whole body was estimated as per equation below:

  $$\begin{array}{c} \text{Retention }\left(\text{\% }\right)=\frac{w2,g\times \text{Mineral in fish at }t_{2},\text{g/kg}-\;w1,g\times \text{mineral in fish at }t_{1},\text{g/kg}}{\left(w2,g-w1,g\right)\times \text{FCR}\times \text{mineral in feed},\text{g/kg}}\times 100, \end{array}$$

where *w* represents weight intervals (initial and final/1 and 2) and *t* represents measured mineral in fish (initial and final/1 and 2).

Throughout the experiment all diets contained yttrium oxide (0.2 g/kgdiet) to measure the apparent digestibility coefficient (ADC), of macro and micro elements. Fecal samples were collected once (Day 146) by dissection of the distal intestine, which is located between the level of the pelvic fins and the anus. From 10 fish per tank, 50 mL fecal samples were collected, weighed and stored at −20°C for further analysis on chemical composition. ADC were determined by following equation [47, 48]:

  $$\begin{array}{c} \text{ADC}=100-100\left(\frac{I_{D}}{I_{F}}\times \frac{N_{F}}{N_{D}}\right), \end{array}$$

where ID represents the yttrium oxide in the diet, IF represents the yttrium oxide in the feces, ND represents the actual nutrient in the diet, and NF represents the actual nutrient in the feces.

The frozen NQCs were defrosted and clean cut, in accordance with NS 9402, so that skin and scales (NQC-skin), muscle (NQC-muscle), and bone (NQC-bone), were separated into individual samples. After separation of each component the individual skin samples were scraped clean with a scalpel to remove the subcutaneous fat, thus, securing a plain NQC-skin sample. The subcutaneous fat was incorporated with the respective NQC-muscle sample. To provide representative samples for the NQC-skin analysis, four 1 cm × 1 cm squares were cut from different part of the NQC-skin sample and prepared separately. These were cut with a knife and fork to avoid Zn contamination from the rubber gloves. From NQC-muscle and NQC-bone, the pooled samples were prepared for each component, with similar amounts of homogenate (min 20 g) from each NQC cut. In the homogenized samples of the whole body, NQC-muscle, and NQC-skin, vitamin D_3_ and the 25(OH)D3 metabolite (calcifediol), were analyzed at Eurofins, by liquid chromatography with diode-array detection (LC_DAD) in accordance with EN12821:2009, while Ca, P, Na, Mg, K, Zn, and Fe were analyzed with ICP-OES in accordance with A120; DS/EN 13805:2014, DS/EN ISO 11885 m:2009. For NQC-bone, minerals were analyzed in addition to ash in accordance with NMKL 173:2005. Sampled feces were freeze-dried. The weight of the samples before and after freeze-drying was measured using gravimetry. Minerals in fecal samples were analyzed as mentioned above in addition to soluble P by Nofima [49].

### 2.6. Statistical Analysis

The statistical software R 4.3.2 was used to analyze all data. For each relevant response, response surface models were fit using generalized linear mixed modeling (through the function lme4::glmer), and considering the two main factors (vitamin D_3_ levels and avP levels in feed). For some responses, additional fixed effects (initial average body weight) and random effects (experimental unit, fish) were also considered. After fitting a series of linear and quadratic models (including a null model), they were compared using the AICc (i.e., Akaike's information criterion with small-sample correction), through the bbmle::ICtab function, in order to select the simplest model that accurately describes the mean response as a function of the relevant factors. For each relevant response, the marginal effects of each factor (as well as 95% confidence intervals [CIs] for the mean response) were then extracted using the emmeans::emmeans function. The *p*-values were obtained by an analysis of deviance comparison between each selected model and the respective null, using a likelihood ratio (chi-square) test. More specifically, the different null models considered were:

  $$\begin{array}{c} \text{SGR}=\beta_{0}+\theta_{\text{fish}}, \end{array}$$

  $$\begin{array}{c} Log_{\text{feed intake}}=\beta_{0}+\beta_{w}\;\log\left(W_{0}\right), \end{array}$$

  $$\begin{array}{c} Log_{FCR}=\beta_{0}+\beta_{w}\;\log\left(W_{0}\right), \end{array}$$

  $$\begin{array}{c} Log_{\text{bone ash}}=\beta_{0}, \end{array}$$

  $$\begin{array}{c} \text{Vitamin D NQC}=\beta_{0}+\;\theta_{\text{unit}}+\beta_{\text{tissue}}\text{tissue}, \end{array}$$

  $$\begin{array}{c} {\text{NQC}}_{\text{tissue}}=\;\beta_{0}+\;\theta_{\text{unit}}+\beta_{\text{tissue}}\text{tissue}, \end{array}$$

where *β*_0_ represents an intercept term, *β*_*w*_ represents the fixed effect of fish size and *β*_tissue_ represents the fixed effect of measuring vitamin D_3_ or minerals in different tissues. Additionally, *θ*_fish_ represents a “fish” random effect, while *θ*_unit_ represents a “unit” random effect.

Based on these null models, a series of increasingly complex models were fit considering additional fixed effects (represented by *β* terms) of (normalized) vitamin D_3_ inclusion levels (vitD) and (normalized) avP inclusion levels, along with their interaction, before proceeding with AICc-based model selection:

  $$\begin{array}{c} \text{Linear }1:\text{ Null model}+\beta_{\text{P}}\text{avP}, \end{array}$$

  $$\begin{array}{c} \text{Linear }2:\text{ Null model}+\beta_{\text{D}}\text{vitD}, \end{array}$$

  $$\begin{array}{c} \text{Linear }3:\text{ Null model}+\beta_{\text{P}}\text{avP}+\beta_{\text{D}}\text{vitD}, \end{array}$$

  $$\begin{array}{c} \text{Quadratic }1:\text{ Null model}+\beta_{\text{P}}\text{avP}+\beta_{\text{D}}\text{vitD}+\beta_{\text{DP}}\text{vitD avP}, \end{array}$$

  $$\begin{array}{c} \text{Quadratic }2:\text{ Null model}+\beta_{\text{P}}\text{avP}+\beta_{\text{D}}\text{vitD}+\beta_{p^{2}}\;{\text{avP}}^{2}, \end{array}$$

  $$\begin{array}{c} \text{Quadratic }3:\text{ Null model}+\beta_{\text{P}}\text{avP}+\beta_{\text{D}}\text{vitD}+\beta_{D^{2}}\;{\text{vitD}}^{2}, \end{array}$$

  $$\begin{array}{c} \text{Quadratic }4:\text{ Null model}+\beta_{\text{P}}\text{avP}+\beta_{\text{D}}\text{vitD}+\beta_{\text{DP}}\text{vitD avP}+\beta_{P^{2}}\;{\text{avP}}^{2}, \end{array}$$

  $$\begin{array}{c} \text{Quadratic }5:\text{ Null model}+\beta_{\text{P}}\text{avP}+\beta_{\text{D}}\text{vitD}+\beta_{\text{DP}}\text{vitD avP}+\beta_{D^{2}}\;{\text{vitD}}^{2}, \end{array}$$

  $$\begin{array}{c} \text{Quadratic }6:\text{ Null model}+\beta_{\text{P}}\text{avP}+\beta_{\text{D}}\text{vitD}+\beta_{\text{DP}}\text{vitD avP}+\beta_{P^{2}}\;{\text{avP}}^{2}+\beta_{D^{2}}\;{\text{vitD}}^{2}. \end{array}$$

## 3. Results

### 3.1. Fish Performance

**Figure 1:**
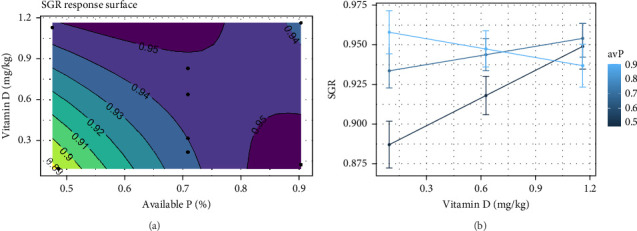
(a) Expected response surface for SGR over the whole design space. The black dots indicate the locations in the design space where measurements were taken. (b) Marginal effects of vitamin D_3_ are a 95% confidence interval for the mean response.

Atlantic salmon with an overall initial weight of 453 ± 9 g and a final weight of 1444 ± 80 g (representing *a* >3-fold increase in body weight) were fed eight different diets. There was no mortality associated with the different dietary treatments throughout the trial period. Feed intake and FCR had the best fit to the null model, indicating no significant effect of different vitamin D_3_ or avP levels (Table 3). In contrast, body weight gain was significantly affected by different inclusion levels of vitamin D_3_ and avP (*p*  < 0.001), supporting the observed interaction effects (Table 3). Increasing vitamin D_3_ when avP was low showed improved SGR. This effect was also identified when avP was increased and vitamin D_3_ was low. When levels of both nutrients were high, the effect was shown as a reduction in SGR (Figure 1A,B). When vitamin D_3_ increased from 0.12 to 0.82 mg/kg, and avP was kept at 0.47% a proportional increase in SGR from 0.88 to 0.93 mg/kg was observed. As vitamin D_3_ increased from 0.82 to 1.12 mg/kg a nonproportional increase in SGR from 0.93% to 0.95% was shown. Increasing avP from 0.47% to 0.66%, while maintaining vitamin D_3_ at 0.12 mg/kg, proportionally increased SGR from 0.88% to 0.93%. The individual effects of an increase in avP levels from 0.67% to 0.90%, resulted in a nonproportional increase in SGR to 0.96%. When vitamin D_3_ and avP increased together, SGR generally rose from 0.88 to a maximum of 0.96%, with the highest SGR result achieved at an intermediate avP inclusion of 0.70%.

### 3.2. Retention and Fecal Excretion

**Figure 2:**
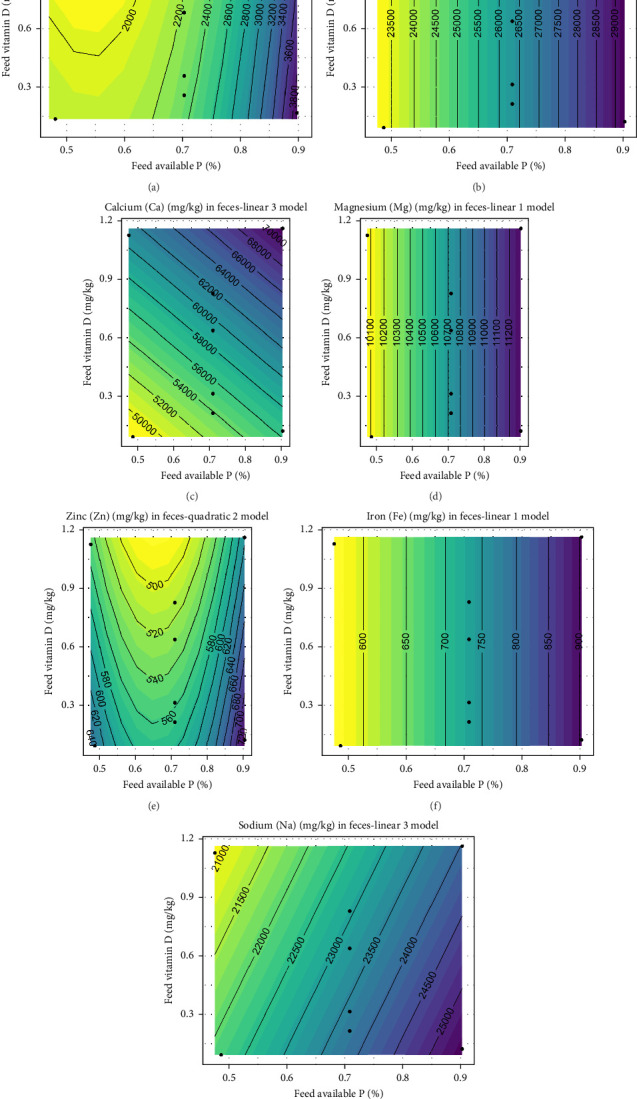
Expected response surface for fecal mineral composition over the whole design space. (a) Soluble phosphorus, (b) Phosphorus (P), (c) Calcium (Ca), (d) Magnesium (Mg), (e) Zinc (Zn), (f) Iron (Fe), and (g) Sodium (Na). The black dots indicate the locations in the design space where measurements were taken.

**Figure 3:**
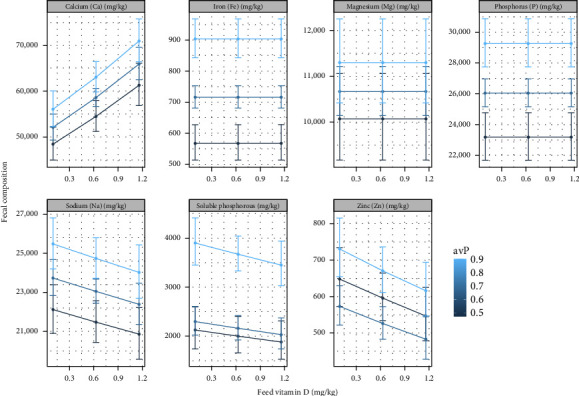
Marginal effects of dietary vitamin D_3_ on fecal composition of minerals, with 95% confidence intervals for the mean response.

A significant effect of different dietary inclusion levels of vitamin D_3_ or avP was observed for all the minerals measured in the fecal samples (Figure 2A–G), though the effect on Mg was nonsignificant according to the likelihood ratio test (*p* = 0.09). Total phosphorus (TP) and Fe indicated an effect only with increasing levels of avP (*p*  < 0.001) and were not influenced by increase in vitamin D_3_. Na decreased in feces with increasing vitamin D_3_ and increased with increased avP (*p*  < 0.001). Soluble P in feces increased as avP increased and decreased as vitamin D_3_ increased (*p*  < 0.001). The decrease in fecal soluble P was more pronounced when dietary avP was high in combination with increased vitamin D_3_. An intermediate level of avP prompted a reduction of approximately 100 mg/kg when increasing vitamin D_3_ from 0.21 to 0.31 mg/kg (Figure 3). Fecal Ca clearly increased both with an increase in vitamin D_3_ and avP (*p*  < 0.001) (Figure 2). Though fecal Zn in low to intermediate inclusions of avP (0.47%–0.70%) was reduced, an increased effect at intermediate to high inclusion levels was shown (*p*  < 0.001). With increasing vitamin D_3_, fecal Zn declined (Figure 3).

**Figure 4:**
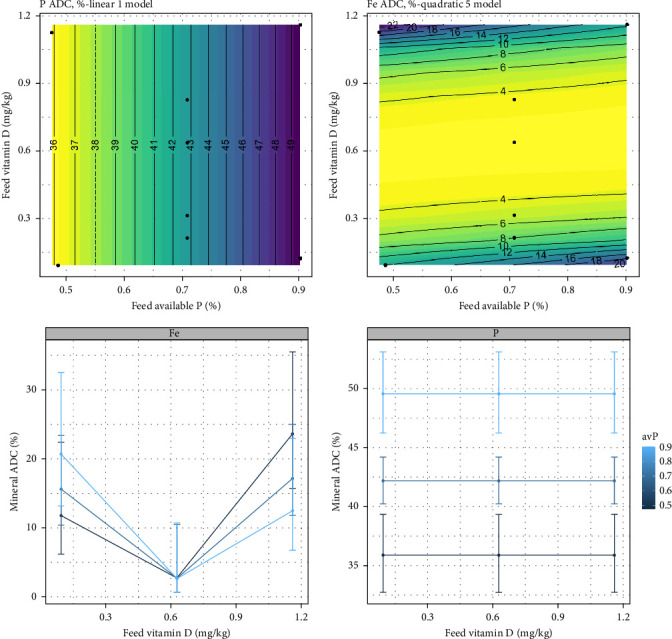
Expected response surface for ADC (%) of iron (Fe) and phosphorus (P) over the whole design space. The black dots indicate the locations in the design space where measurements were taken. Marginal effects of dietary vitamin D_3_, with 95% confidence intervals for the mean response.

The ADC of Fe declined for the fish-fed intermediate levels of vitamin D_3_ (*p*  < 0.001). The combined effect of vitamin D_3_ and avP was minimal. No effect was observed for ADC of Zn. ADC of TP did not show any effect at increasing vitamin D_3_ levels but had a clear trend to increase with increased avP (*p*  < 0.001) (Figure 4).

**Figure 5:**
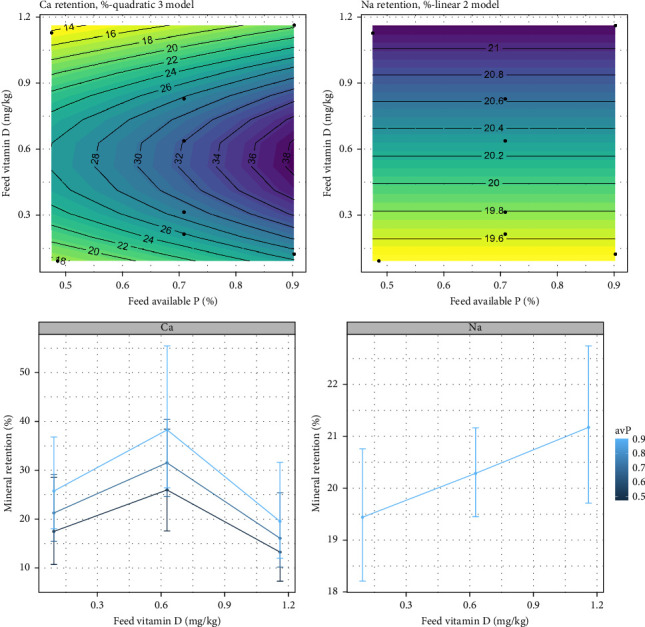
Expected response surface for calcium (Ca) and sodium (Na) retention over the whole design space. The black dots indicate the locations in the design space where measurements were taken. Marginal effects of vitamin D_3_ on mineral retention, with 95% confidence intervals for the mean response.

For whole body retention, no effects of the dietary treatments were observed for Zn, Mg, TP, or Fe. A tendency for increased Na retention was observed with increasing vitamin D_3_, where Na retention increased proportionally from 19% to 21% with vitamin D_3_ inclusion of 0.09–1.12 mg/kg in the diet (Figure 5), though this trend was nonsignificant according to the likelihood ratio test (*p* = 0.10). The Ca retention responded both to vitamin D_3_ and avP (*p*  < 0.05), with a proportional increase in retention observed at increasing avP from 0.47% to 0.90% (Figure 5). An increase in vitamin D_3_ from low to intermediate levels (0.09–0.63 mg/kg) promoted an increased Ca retention from 25% to 32% (Figure 5). The results indicate that increasing both vitamin D_3_ and avP can increase Ca retention.

**Figure 6:**
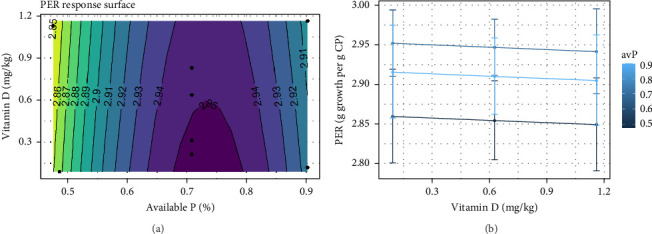
(a) Expected response surface for protein efficiency ratio (PER) over the whole design space. The black dots indicate the locations in the design space where measurements were taken. (b) Marginal effects of vitamin D_3_ on protein efficiency ratio, with 95% confidence intervals for the mean response.

Although a slight decrease was observed in PER as vitamin D_3_ levels increased, the main influence on PER was observed when avP changed (*p*  < 0.05; Figure 6A,B). Increasing avP in the diet from low to intermediate levels (0.47%–0.90%) was shown to influence PER through an increase from 2.85 to 2.95. An increase in avP above this level showed a decrease in PER.

### 3.3. Whole Body Composition

**Figure 7:**
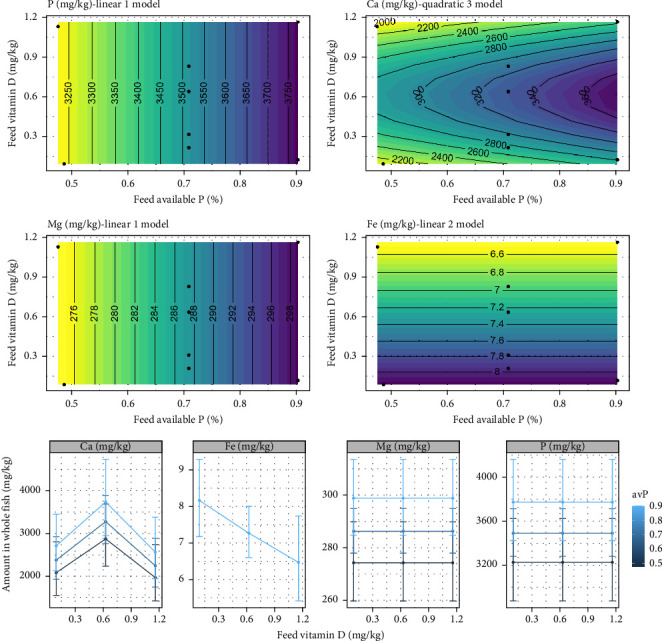
Expected response surface for whole body mineral composition over the whole design space. The black dots indicate the locations in the design space where measurements were taken. Only non-null models are displayed. Marginal effects of each factor on whole fish mineral composition, with 95% confidence intervals for the mean response.

The whole-body TP and Mg showed no relationship with dietary vitamin D_3_. Increasing levels of avP in the diet of 0.47%–0.90% resulted in a possible increase, in the whole-body TP, increasing from 3250 to 3750 mg/kg (*p* = 0.06), whilst Mg significantly increased from 276 to 298 mg/kg (*p*  < 0.05). In contrast, there was no effect of avP on whole body Fe, though, increased whole body Fe was observed with increasing vitamin D_3_ (*p*  < 0.05). No effect on either vitamin D_3_ or avP were observed for Zn or Na. Increasing both vitamin D_3_ and avP had a significant effect on the Ca level (*p*  < 0.05), whereby an increased level of vitamin D_3_ above 0.6 mg/kg caused a clear reduction in whole body Ca (Figure 7).

### 3.4. NQC Tissue Analysis

**Figure 8:**
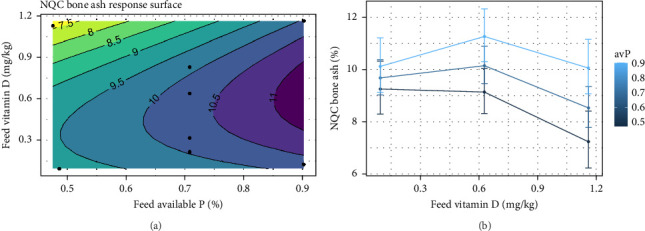
(a) Expected response surface for NQC bone ash over the whole design space. The black dots indicate the locations in the design space where measurements were taken. 95% CI for the mean response. (b) Marginal effects of each factor on NQC bone ash in Atlantic salmon (*Salmo salar*), with 95% confidence intervals for the mean response.

An increase in avP had a positive effect on bone ash content analyzed from the NQC, where an increase by 2% was observed from low to high inclusions of avP (Figure 8A). In contrast, by only increasing vitamin D_3_ in the diet, a reduction in bone ash content from 9% to 7.5% was observed (Figure 8B). As vitamin D_3_ increased above 0.6 mg/kg and avP also increased, there is a reduction in bone ash, with the largest effect observed when Vitamin D_3_ is high and avP is low (Figure 8A). This interaction was evident across intermediate and high avP inclusions (*p*  < 0.001). When vitamin D_3_ is increased from low to intermediate levels, there is a positive effect on bone ash at both intermediate and high levels of avP, with the largest effect observed at 0.9% avP.

**Figure 9:**
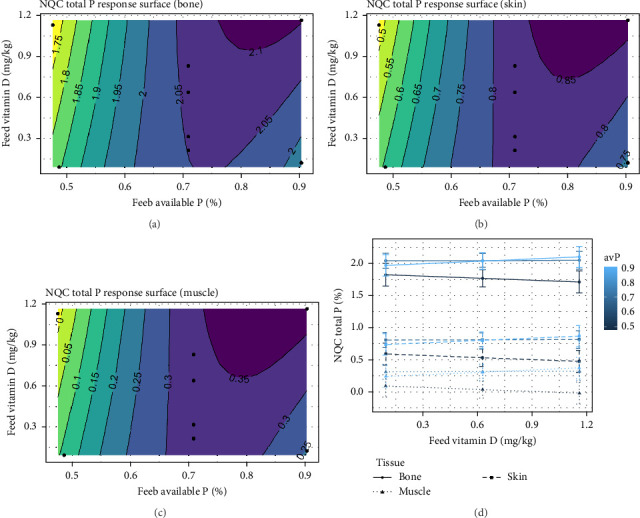
Expected response surface for NQC total phosphorus (TP) over the whole design space, either in the bone (a), in the skin (b), or in the muscle (c). The black dots indicate the locations in the design space where measurements were taken. (d) Marginal effects of each factor on NQC TP, with 95% confidence intervals for the mean response.

For NQC TP in different tissue, an effect was observed with combined levels of vitamin D_3_ and avP in the diet (*p*  < 0.001). The NQC bone, skin and muscle could be maintained at 2.05% (Figure 9A), 0.80% (Figure 9B), and 0.30% (Figure 9C) of tissue weight respectively, with vitamin D_3_ levels at 0.21 upto 0.70 mg/kg and avP levels at 0.68%–0.81%. The effect of vitamin D_3_ alone on accumulation in tissue was however low (Figure 9D). A proportional increase in NQC TP for all tissues was observed when avP rose from low to intermediate levels.

**Figure 10:**
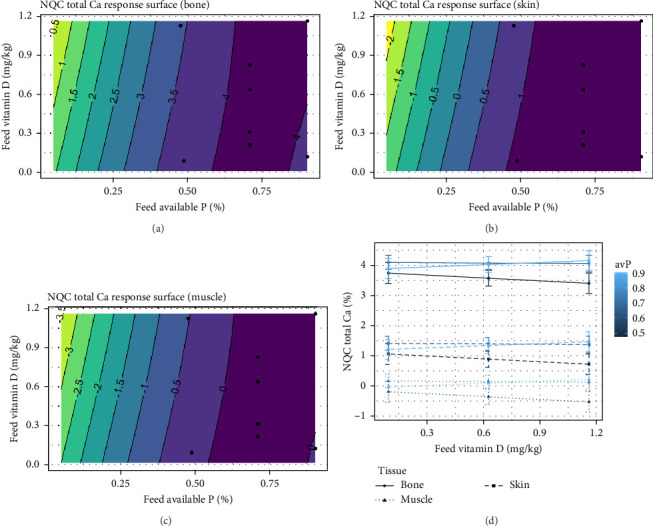
Expected response surface for NQC calcium (Ca) over the whole design space, either in the bone (a), in the skin (b), or in the muscle (c). The black dots indicate the locations in the design space where measurements were taken. (d) Marginal effects of each factor on NQC total Ca, with 95% confidence intervals for the mean response.

**Figure 11:**
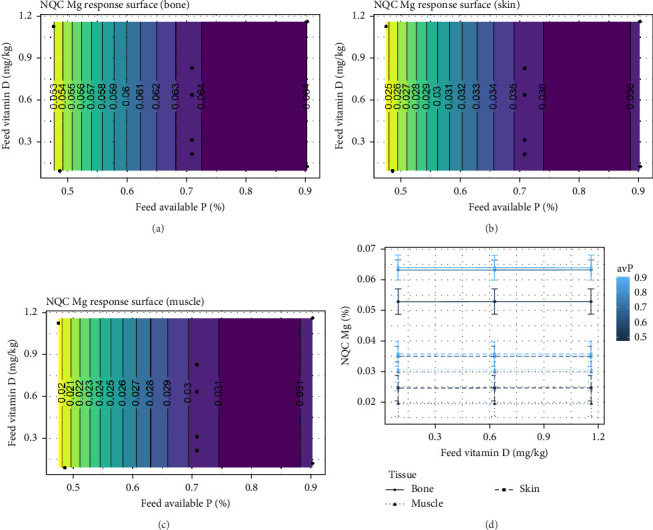
Expected response surface for NQC magnesium (Mg) content over the whole design space, either in the bone (a), in the skin (b), or in the muscle (c). The black dots indicate the locations in the design space where measurements were taken. (d) Marginal effects of each factor on NQC magnesium content, with 95% confidence intervals for the mean response.

The NQC Ca showed similar response in muscle and bone, and at intermediate dietary inclusion of avP, no response was expected for NQC Ca when vitamin D_3_ increased from 0.21 to 1.12 mg/kg (*p*  < 0.001) (Figure 10A–D). While the NQC Mg showed no effect of vitamin D_3_, a strong effect of avP was observed (*p*  < 0.001; Figure 11A–D). No effect of either avP or vitamin D_3_ were observed for Zn or Na.

### 3.5. NQC Vitamin D_3_

**Figure 12:**
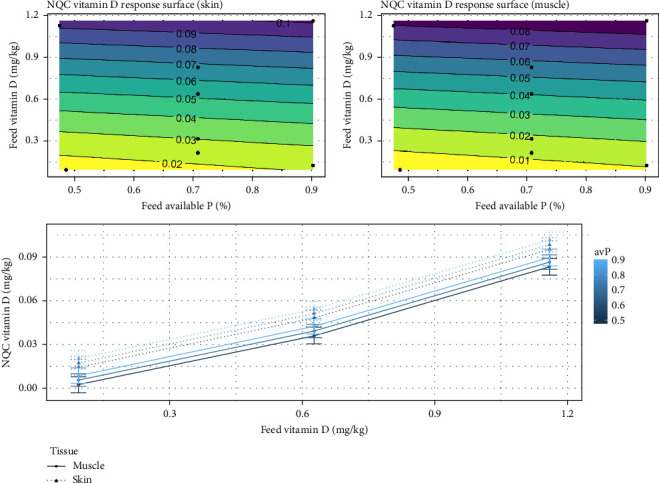
Expected response surface for tissue vitamin D_3_ over the whole design space, either in the skin (top plot), or in the muscle (bottom plot). The black dots indicate the locations in the design space where measurements were taken. Marginal effects of each factor on NQC vitamin D_3_, with 95% confidence intervals for the mean response.

The modeled results of vitamin D_3_ in NQC, showed that increasing vitamin D_3_ levels from 0.21 to 1.2 mg/kg increased vitamin D_3_ levels in the skin by 500% (0.015–0.090) (Figure 12). Increasing only avP levels had a marginal effect on vitamin D_3_ levels in both skin and muscle. This effect was also observed when both avP and vitamin D_3_ were increased proportionally (*p*  < 0.001).

## 4. Discussion

The dietary requirement of vitamin D_3_ varies depending on fish species, environment, and life stage [1, 50]. However, there is a limited number of studies exploring the requirement for vitamin D_3_ in cultivated salmonids. The current study aimed to explore the metabolic responses to different dietary levels of vitamin D_3_ and P in farmed Atlantic salmon. Growth performance was influenced by the interaction between vitamin D_3_ and avP, with independent increases improving SGR, while simultaneous elevation to the highest inclusion led to a slightly reduced growth. This nonlinear interaction effect on SGR at the highest inclusion levels are inconsistent with pervious patterns and remains difficult to interpret. Additional investigation is required to gain further insight.

The individual effects of the current results are, however, comparable with several studies across different species, including Siberian sturgeon (*Acipenser baerii*), Nile tilapia (*Oreochromis niloticus*), channel catfish (*Ictalurus punctatus*), and rainbow trout, which all have demonstrated improved growth performance with increasing dietary vitamin D_3_, though the effective dose varies by species and life stage [51–54]. Both low and excessive (62.5 mg/kg) amounts of dietary inclusion of vitamin D_3_ have been shown to reduce growth in rainbow trout [16, 54]. The benefit of increased vitamin D_3_ to growth, may be explained by its effect on fatty acid oxidation, where it has been demonstrated that increasing calcitriol (1.25(OH)_2_D_3_) and calcifediol in zebrafish and juvenile Atlantic salmon, can improve lipid metabolism [55, 56]. However, it should be noted that in the present study, all calcifediol measurements were below the lower limit of quantification, which limits direct interpretation of its contribution to the observed growth effects. Lipids are a dense energy source and when fatty acid oxidation is upregulated, more adenosine triphosphate is produced, which fuels processes, like protein synthesis, cell division, and tissue development. Calcitriol and calcifediol is also known to enhance the insulin pathway regulation and insulin activity, which additionally can contribute to an improved growth performance in zebrafish [57, 58]. Increasing calcifediol in diets for juvenile Atlantic salmon has been shown to improve FCR [56]. Similarly, in rainbow trout, increasing vitamin D from 0.13 to 0.82 mg/kg through calcifediol addition resulted in improved SGR and FCR, but did not lead to a noteworthy increase in total vitamin D concentration in the filet [59]. Based on the current and previous studies, it may be beneficial to increase vitamin D in diets with higher lipid levels when cultivating salmonid species, for example, for commercial RAS diets that typically have higher lipid levels compared to traditional diets [33].

Whilst many P requirement studies in freshwater-reared salmonid species do not report a significant increase in growth with increasing dietary P levels [60–63], there have been observations of increased growth for Atlantic salmon fed 9400 mg/kg TP compared to 7100 and 16,300 mg/kg [64], which is similar to what was observed in the current study. Deficient P diets have consistently led to reduced growth across salmonid species in both freshwater and seawater [65–68], and in the present study, the LP diets (e.g., 0.476% avP) fell slightly below the NRC recommendations, resulting in possibly reduced bone ash and whole-body P (*p* = 0.06), which suggests the possibility of a marginal deficiency. However, these effects did not translate into significant changes in FCR or PER, suggesting that the putative deficiency was subclinical rather than overt. While hypophosphatemia was not directly assessed in this study and cannot be confirmed, the results highlight the importance of precise dietary formulation, especially when optimizing vitamin D_3_ and P interactions for growth and mineralization.

These findings suggests that while low avP diets may not overly impair feed efficiency, they can subtly affect growth. Building on this, the current study's two-level full factorial design with additional points revealed a modest approximately 2% improvement in SGR when vitamin D_3_ was increased from 0.21 to 1.12 mg/kg at intermediate avP levels (0.63 %). This contrasts with earlier studies reporting no significant interaction between vitamin D_3_ (0.06, 6.25, and 62.50 mg/kg) and P in relation to growth [15, 16], potentially due to the differences in experimental design. The response surface approach used here allowed detection and characterization of nonlinear responses that a 2 × 2 or a 2 × 3 factorial design would not be able to detect. The initial increases in vitamin D_3_ and avP lead to proportional increases in SGR, but further increases resulted in smaller, nonproportional gains, which is a nonlinear observation. Additionally, the curvature showed that there were optimal ranges for vitamin D_3_ and avP where the SGR was maximized, and that deviations from these ranges resulted in reduced SGR. Even though the observed effect was very modest, it is possible to enhance growth performance of Atlantic salmon while staying within the EU regulation limit of 1.5 mg/kg [23].

In addition to vitamin D and P levels, the dietary Ca:P ratio may also influence mineralization outcomes and trace mineral absorption, where low Ca:P ratio generally reduce both growth and Zn absorption [69, 70]. In a P deficient state, fish may for instance compensate by excreting excess Ca to stabilize plasma P, resulting in reduced whole-body Ca and P and a lower Ca:P ratio [67, 71, 72]. Although the diets in the current study were only regulated on vitamin D_3_ and avP, higher P concentrations (e.g., HP-LD) resulted in slightly lower Ca:P ratios compared to the diet with lower P concentrations (LP-HD). However, all the experimental diets maintained a Ca:P ratio near 1.0, which is suggested as optimal [24, 69], with the HP-LD and LP-HD ranging from 0.7 to 1.4, respectively, with the HP-LD diet displaying the best SGR performance. Whole body Ca:P ratio in the current study was within the expected ranges for Atlantic salmon adults in seawater (1–4 kg), previously reported at 0.7–0.8 [73] and the bone and NQC skin samples (~2.0), and within the expected ratio for rainbow trout in freshwater at 1.92–2.09 [73]. Additionally, ash, P, Ca, and Mg concentrations in whole body and NQC tissues were within seawater-reared ranges [67]. The lower coefficient of variation in Ca:P ratio (18%) compared vitamin D_3_ (78%) and avP (23%), further suggests that Ca:P was not a major driver of the observed effects.

The reduction in bone ash with increasing dietary vitamin D_3_, particularly in the LP diets, may indicate a shift toward bone resorption rather than mineralization, potentially driven by endocrine regulation of P. Under low plasma phosphate, calcitriol synthesis is upregulated to enhance intestinal P absorption [74], and elevated plasma levels of calcitriol and calcifediol have been reported in Atlantic salmon fed low levels of P (6300 and 4000 mg/kg avP), in contrast to salmon fed higher levels of P [75, 76]. Elevated plasma calcitriol levels observed in Atlantic salmon and rainbow trout fed LP diets [15, 62, 75] suggest a compensatory endocrine response aimed at maintaining mineral homeostasis.

When dietary P is limiting, fish may activate compensatory mechanisms to maintain mineral homeostasis. In seawater environments, where Ca is abundant, these mechanisms may be further modulated by environmental factors. Calcitriol can inhibit osteoblast activity and promote osteoclast-mediated bone demineralization [77], mobilizing skeletal stores to stabilize plasma Ca and P. Additionally, seawater adaptation in rainbow trout alters vitamin D metabolite binding in intestinal tissues, with increased 24,25(OH)_2_D_3_ and reduced calcitriol binding, correlating with suppressed Ca uptake [78]. This shift may increase reliance on skeletal Ca, especially when dietary P is limiting. Although vitamin D_3_ typically enhances Ca absorption via CaBP upregulation in the intestine [79], this pathway may be less effective under conditions of marginal P deficiency or altered metabolic regulation. Hayes et al. [80] demonstrated that rainbow trout adapted to seawater or high-Ca environments preferentially metabolized calcifediol into 25,26(OH)_2_D_3_ rather than calcitriol, and that intestinal Ca uptake may be suppressed in fasted fish despite elevated calcitriol levels. This suggests that nonintestinal pathways or skeletal mobilization may dominate under LP conditions, particularly in seawater-adapted fish. This hypothesis aligns with the current results where the fish were reared at 28 psu salinity (which provides a Ca rich environment [24]), fecal TP remained stable, and fecal Ca increased with higher dietary vitamin D_3_. Whole-body Ca and Ca retention increased up to 0.63 mg/kg vitamin D_3_, but declined at higher inclusion levels, suggesting a threshold effect.

Although a freshwater study, a similar threshold effect has been observed in European sea bass larvae (*Dicentrarchus labrax*), where dietary vitamin D_3_ levels just 0.35–0.40 mg/kg above or below the optimal dose significantly increased malformations [81]. In contrast, teleosts such Atlantic salmon and rainbow trout appear to tolerate a wider range of dietary vitamin D_3_. This may be due to their ability to metabolize vitamin D_3_ into less active forms like 24,25(OH)_2_D_3_ that has a suppressive effect on Ca absorption in seawater adapted salmon similar to marine fish [82, 83]. Graff et al. [82] reported low circulating calcifediol levels and high production of this metabolite, in seawater adapted Atlantic salmon suggesting a regulatory mechanism that buffers against excess vitamin D_3_ [82, 84]. Notably, calcitriol has also been shown to enhance Zn uptake in rainbow trout gills via upregulation of the Zn transporter protein ZIP1 and ECaC (a Ca channel responsive to calcitriol that also facilitates Zn transport) [85]. This regulatory mechanism has not previously been discussed in the context of seawater adaptation or diets low in P, making the current findings novel in linking vitamin D_3_-mediated Zn retention to environmental and dietary stressors. Although no reduction in whole-body Zn was detected, the observed 11% decrease in fecal Zn with increasing dietary vitamin D_3_, suggests reduced Zn excretion, consistent with endocrine-driven mobilization and conserved of essential minerals under LP conditions. These findings support the hypothesis that vitamin D_3_ acts on shared regulatory pathways for Ca and Zn, and that seawater adaptation and LP conditions may trigger endocrine responses that affect both minerals through coordinated transport and mobilization mechanisms. Since the exact role of these different vitamin D metabolites remain unclear [84, 86], further research is required.

Fecal concentrations of P in the current study ranged from 2.3%–3.0% DW and was primarily influenced by the digestibility and availability of dietary sources, rather than metabolic regulation [32, 87–89]. As dietary MAP increased, total fecal P rose steadily, while fecal soluble P showed a sharper increase above 0.63% avP, suggesting that dietary intake exceeded metabolic requirements [24, 35, 90]. This pattern likely reflects unabsorbed dietary P and endogenous P excretion, rather than regulatory losses, which are typically associated with renal pathways [88]. The steep rise in soluble fecal P may indicate saturation of intestinal absorption mechanisms and passive excretion of excess P.

In an earlier study on rainbow trout it was observed that increasing vitamin D_3_ from 0.06 to 0.25 mg/kg in the diet could cause a reduction in fecal TP [32]. Whilst the current study did not observe a similar reduction in fecal TP, it did reveal a approximately 9% decrease in fecal soluble P when vitamin D_3_ was increased from today's commercial inclusion levels of approximately 0.21–1.12 mg/kg. This observation is highly relevant for waste management from aquaculture facilities, since the soluble P in feces can more easily leach into the water making it harder to collect and purify in traditional mechanical waste water treatment [34, 49, 91]. Additionally, the decline in fecal Zn concentrations of approximately 11% with increasing vitamin D_3_, suggests a broader role for vitamin D in minimizing mineral excretion, which can be valuable as Zn is a limiting factor for the further utilization of aquaculture sludge, for example, fertilizer for agriculture [92]. These findings highlight the potential of using vitamin D_3_ not only to enhance growth but also to reduce environmental discharge of key nutrients. Strategic increases in vitamin D_3_ may allow for modest reductions in avP without compromising performance, provided that SGR (≥0.94%) and bone ash content (>10%) are maintained (Figure 1A,B). However, excessive reductions in avP could impair growth and increase feed requirements, potentially offsetting the environmental benefits. Therefore, careful balancing of vitamin D_3_ and avP is recommended to optimize both fish performance and sustainability in land-based salmon farming.

In accordance with previous studies, this study demonstrated that Atlantic salmon, responded to vitamin D_3_ in the diet and deposited the vitamin in the fillet and skin by accumulating vitamin D_3_ in a dose dependent manner similarly in the tissues investigated [29, 93]. Although the vitamin D_3_ in all diets for the current study were within the recommended levels for Atlantic salmon [94], the analyzed concentration ranged from 0.78 to 8.34 µg/100 g. Notably, fish-fed the HP-LD, LP-LD, MP-CD, and MP-D03 diets had muscle concentrations below the expected commercial range of 2.9–9.5µg/100 g [28]. The control diet (MP-CD), containing 0.21 mg/kg vitamin D_3_, resulted in an average muscle deposition of only 1.53 ± 0.41 µg/100 g vitamin D_3_. These levels from fish reared indoors without exposure to natural light is significantly lower than the NQC average of 10 µg/100 g vitamin D_3_ found in Atlantic salmon reared in open net pens with natural light exposure in Norway during June [95].

Farmed Atlantic salmon generally contain lower levels of vitamin D_3_ in the filet compared to wild salmon, with wild North sea salmon averaging 9.4 ± 1.9 µg/100 g and wild Baltic sea salmon 18.5 ± 4.6 µg/100 g [28]. Data from over 1000 NQC's collected between 2006–2023 show an average vitamin D_3_ concentration of 7.7 µg/100 g in farmed Atlantic salmon filet [45, 96], while wild salmon averaged 8.9 µg/100 g. This discrepancy highlights the nutritional implications for human consumers, as farmed salmon are marketed as a source of vitamin D_3_. These findings suggest that land-based systems may require higher dietary vitamin D_3_ inclusion to maintain filet concentrations that meet nutritional expectations and labeling standards. Given that the recommended daily intake of vitamin D_3_ for human adults is 15 µg [97], with an increase in levels of vitamin D_3_ in aquafeeds for salmon to 0.63 mg/kg, the salmon filet may contribute only approximately 24% of that amount per 100 g serving or 3.6 µg, still well within the safety limit. This contribution is close to the average daily intake of vitamin D from fish and fish products in Finland (4.4 µg/day per capita), suggesting that even modest increases in dietary vitamin D_3_ in aquafeeds can significantly support population-level intake through regular consumption of farmed salmon [98]. Although dietary intake remains the primary source of vitamin D_3_ in fish [1, 25–27, 99], the findings from this trial suggest a potential association between light exposure and tissue-level deposition of vitamin D_3_, indicating that photochemical synthesis may play a supplementary role under certain rearing conditions. Though this hypothesis has not been tested in the current trial, further research should investigate the potential for vitamin D_3_ synthesis from light exposure in Atlantic salmon reared in seawater, including the effects of light spectrum, intensity and photoperiod. Additionally, a cost-benefit analysis comparing investment in light technology versus increased dietary vitamin D_3_ inclusion could help determine the most efficient strategy for maintaining nutritional quality in closed aquaculture system. This information would be highly relevant with the expected growth planned for production of salmon in closed aquaculture farming that use artificial or low light [100].

## 5. Conclusion

This study demonstrates a nuanced interaction between dietary vitamin D_3_ and avP in Atlantic salmon post-smolts reared in land-based systems. While increased vitamin D_3_ inclusion (upto 1.12 mg/kg) improved SGR under certain avP conditions, excessive levels of both nutrients led to reduced bone ash and whole-body Ca, suggesting a threshold beyond which mineralization may be compromised.

The optimal dietary combination was identified at 0.63 mg/kg vitamin D_3_ and 0.70% avP which supported satisfactory growth and mineral retention, while remaining within EU regulatory limits (max 1.5 mg/kg vitamin D_3_). This clearly show that it is possible to achieve satisfactory filet levels of vitamin D_3_ while being well within safe inclusion levels regarding human consumption.

Importantly, increasing vitamin D_3_ reduced soluble phosphorus in feces by approximately 9%, which may contribute to lower environmental P discharge. Although not modeled in this study, this reduction could translate to improved waste management efficiency in RAS, and other systems where mechanical filtration is used.

Finally, salmon reared without natural light exposure accumulated lower vitamin D_3_ in muscle compared to reported levels for salmon raised in open-pen, reinforcing the need to adjust dietary vitamin D_3_ levels in closed systems to maintain nutritional quality for human consumption.

## Figures and Tables

**Table 1:** Formulation of experimental diet (%).

Ingredient	LP-HD	HP-HD	HP-LD	LP-LD	MP-MD	MP-CD	MP-D03	MP-D08
Fish meal^a^	24.3	24.4	24.5	24.2	24.4	24.4	24.4	24.4
SPC^b^	12.0	11.7	11.7	11.9	11.6	11.8	11.7	11.7
PPC^c^	6.0	6.0	6.0	6.0	6.0	6.0	6.0	6.0
Wheat gluten^d^	10.0	10.0	10.0	10.0	10.0	10.0	10.0	10.0
Raw wheat	9.7	9.7	9.7	9.7	9.7	9.7	9.7	9.7
Corn gluten^e^	2.0	2.3	2.1	2.2	2.3	2.1	2.2	2.4
Pea hulls^f^	0.7	0	0.6	0.9	0.1	0.5	0.4	0.0
Faba, dehulled	2.0	2.0	2.0	2.0	2.0	2.0	2.0	2.0
Guar meal^g^	2.0	2.0	2.0	2.0	2.0	2.0	2.0	2.0
Rapeseed oil	22.5	22.5	22.5	22.5	22.5	22.5	22.5	22.5
Algae oil^h^	5.8	5.8	5.8	5.8	5.8	5.8	5.8	5.8
MAP^i^	0	1.7	1.7	0	0.8	0.8	0.8	0.8
Vitamin D_3_ premix^j^	0.7	0.7	0	0	0.4	0.04	0.2	0.5
Microingredients^k^	1.9	1.9	1.9	1.9	1.9	1.9	1.9	1.9
Water balance	0.4	−0.7	−0.5	0.9	0.5	0.4	0.4	0.3

*Note:* The relative amount of each microingredient was fixed across all diets and supplied according to standard commercial aquafeed practices.

Abbreviations: MAP, monoammonium phosphate; PPC, pea protein concentrate; SPC, soy protein concentrate.

^a^Norse LT94: 71.3% CP, 9.4% CF, 14.6% ash, 7.3% water; Pelagia, Egersund, Norway.

^b^Soy protein concentrate: 61.8% CP, 0.4% CF, 6.7% ash, 7.5% water; CJ Selecta, Araguari, Brazil.

^c^Pea protein concentrate: 76.4% CP, 3.8% CF, 1.6% ash, 10.6% water.

^d^Wheat gluten: 75.0% CP, 1.1% CF, 1.2% ash, 9.4% water.

^e^Provided by ADM Razgard EAD, Razgard, Bulgaria.

^f^Provided by AM Nutrition, Stavanger, Norway.

^g^Provided by Meghraj International, Haryana State, India.

^h^AlgeaPrime-Core, provided by Corbion, Amsterdam, Netherlands.

^i^MAP, provided by Yara Norway AS, Drammen, Norway.

^j^Provided by DSM-Firmenich, Kaiseraugst, Switzerland.

^k^Dry fat, choline-inositol mix, L-lysine 78%, methionine, mineralmix EWOS, L-threonine, lucantin pink 10% SD CWS, histidine, vitamin premix, vitamin C 35%, and vitamin E 50%.

**Table 2:** Measured proximate composition, vitamin D_3_, and P content of experimental diets.

Parameter	LP-HD	HP-HD	HP-LD	LP-LD	MP-MD	MP-CD	MP-D03	MP-D08
Proximal composition (%)								
Protein	43.8	43.7	43.2	43.8	44.4	42.5	43.8	43.3
Fat	29.7	30.5	30.4	30.6	30.5	31.0	30.9	30.7
Moisture	7.0	6.8	6.5	6.6	6.4	6.3	7.1	6.9
Gross energy (MJ/kg)	24.08	24.17	24.44	24.27	24.15	24.57	24.37	24.31
Ash	5.5	5.7	5.2	4.6	5.5	5.5	5.8	5.4
Total phosphorus	0.85	1.30	1.30	0.87	1.10	1.10	1.10	1.10
Available phosphorus^a^	0.476	0.902	0.902	0.487	0.708	0.708	0.708	0.708
Calcium	1.20	1.30	0.97	1.00	1.10	1.10	1.10	1.20
Magnesium	0.16	0.17	0.18	0.16	0.17	0.17	0.17	0.17
Sodium	0.32	0.34	0.34	0.35	0.34	0.35	0.35	0.34
Zn	0.019	0.019	0.023	0.020	0.015	0.020	0.020	0.019
Fe	0.017	0.021	0.026	0.015	0.016	0.019	0.018	0.017
Vitamin D_3_ (mg/kg)	1.125	1.160	0.125	0.094	0.637	0.215	0.315	0.827

^a^Estimated based on internal Cargill data obtained through raw material digestibility and retention studies.

**Table 3:** Fish performance parameters in Atlantic salmon (*Salmo salar*) at different dietary treatments of vitamin D_3_ and avP.

Parameter	Diet							
	LP-HD	HP-HD	HP-LD	LP-LD	MP-MD	MP-8 kD	MP-16 kD	MP-46 kD
Body weight gain (g)								
Modeled mean response (±SE)	1484 ± 10	1467 ± 9	1503 ± 9	1387 ± 9	1481 ± 5	1470 ± 5	1472 ± 5	1486 ± 6
Modeled mean (lower 95% CI)	1465	1449	1485	1369	1471	1459	1462	1475
Modeled mean (upper 95% CI)	1504	1485	1521	1406	1491	1480	1482	1497
Feed intake (kg)								
Raw response (±SD)	24.7 ± 2.96	25.4 ± 0.47	26.3 ± 0.97	23.3 ± 0.35	25.0 ± 1.80	24.6 ± 1.89	26.1 ± 1.79	24.7 ± 1.12
FCR								
Raw response (±SD)	0.80 ± 0.01	0.79 ± 0.01	0.80 ± 0.02	0.81 ± 0.02	0.78 ± 0.01	0.79 ± 0.02	0.78 ± 0.01	0.78 ± 0.01

*Note:* Data are expressed as averages on fish basis ± SE for each dietary treatment with a 95% confidence interval.

Abbreviations: CI, confidence interval; FCR, feed conversion ratio.

## Data Availability

The data presented in this study are available on request from the corresponding author due to privacy restrictions.

## Nomenclature

avP: Available phosphorus
Ca: Calcium
FCR: Feed conversion rate
Fe: Iron
MAP: Monoammonium phosphate
Mg: Magnesium
NQC: Norwegian quality cut
P: Phosphorus
PER: Protein efficiency ratio
RAS: Recirculating aquaculture system
SGR: Specific growth rate
TP: Total phosphorus
Zn: Zinc
